# Interference Range-Reduced Cooperative Multiple Access with Optimal Relay Selection for Large Scale Wireless Networks

**DOI:** 10.3390/s19112565

**Published:** 2019-06-05

**Authors:** Kai Liu, Rui Wang, Caizhao Yue, Feng Liu, Tao Lu, Zixiang Xiong

**Affiliations:** 1 School of Electronics and Information Engineering, Beihang University, Beijing 100191, China; liuk@buaa.edu.cn (K.L.); julieruiwang@gmail.com (R.W.); yuecaizhao@foxmail.com (C.Y.); liuf@buaa.edu.cn (F.L.); 2 Beijing Laboratory for General Aviation Technology, Beijing 100191, China; 3 Beijing Key Laboratory for Network-Based Cooperative Air Traffic Management, Beijing 100191, China; 4 Sino-French Engineer School, Beihang University, Beijing 100191, China; 5 School of Computer Science and Engineering, Wuhan Institute of Technology, Wuhan 430073, China; 6 Department of Electrical and Computer Engineering, Texas A&M University, College Station, TX 77840, USA; zx@ece.tamu.edu

**Keywords:** large scale wireless networks, medium access control (MAC), cooperative MAC protocol, helper selection, spatial diversity gain, spatial frequency reuse gain

## Abstract

Cooperative communication improves the link throughput of wireless networks through spatial diversity. However, it reduces the frequency reuse of the entire network due to the enlarged link interference range introduced by each helper. In this paper, we propose a cooperative medium access control (MAC) protocol with optimal relay selection (ORS-CMAC) for multihop, multirate large scale networks, which can reduce the interference range and improve the network throughput. Then, we investigate the performance gain achieved by these two competitive factors, i.e., the spatial frequency reuse gain and spatial diversity gain, in large scale wireless networks. The expressions of maximum network throughput for direct transmissions and cooperative transmissions in the whole network are derived as a function of the number of concurrent transmission links, data packet length, and average packet transmission time. Simulation results validate the effectiveness of the theoretical results. The theoretical and simulation results show that the helper can reduce the spatial frequency reuse slightly, and spatial diversity gain can compensate for the decrease of the spatial frequency reuse, thereby improving the network throughput from the viewpoint of the whole network.

## 1. Introduction

Large scale wireless networks, such as mobile ad hoc networks, wireless sensor networks, and vehicle ad hoc networks, have attracted increasingly more interest in the past decade because of their easy deployment and fast configuration [1]. However, fading, path loss, and shadowing problems severely affect their throughput and transmission reliability. Although multiple-input, multiple-output (MIMO) can effectively solve the above problems [2], it is not feasible to integrate several antennas within a small size, portable, and battery-powered device because of the limit of size and energy consumption. Cooperative communication can achieve the virtual antenna arrays via the neighboring nodes in multihop wireless networks, and therefore it is an effective solution to address the problems of resource constraint and channel fading [3].

Early research work on cooperative communication techniques focused on the physical (PHY) layer [4,5,6,7], in which one or more helpers is used to explore the spatial diversity gain (SDG) to enhance the transmission reliability, increase the transmission rate between sender-recipient pairs, and reduce the packet transmission delay. However, the interaction with the upper layer is usually ignored. In order to fully utilize the cooperation gain generated in the PHY layer, increasingly more research work has applied cooperative communication to higher layers, especially the adjacent medium access control (MAC) layer. The MAC protocol is used to solve the medium sharing problem among multiple nodes, which is critical to network performance, such as throughput and delay. A well-designed cooperative MAC (CMAC) protocol should not only consider the instantaneous channel quality and minimize the overhead required by cooperative communication at the MAC layer, but also take the application environment into account. Otherwise, its effectiveness and efficiency can be greatly reduced.

**Figure 1:**
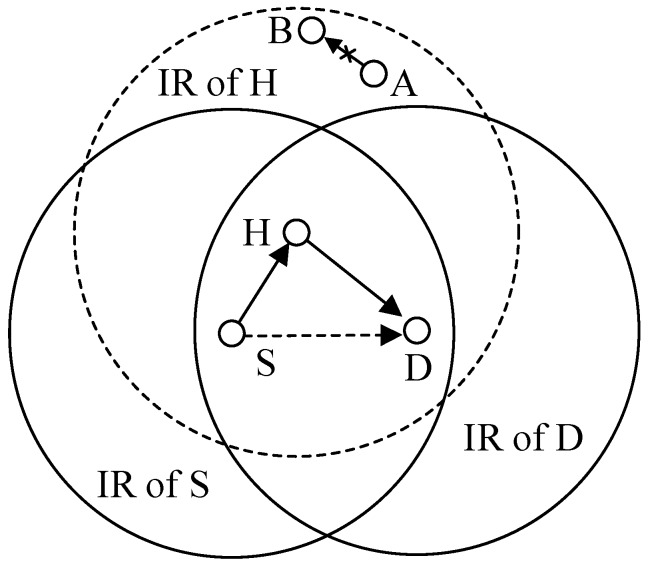
Enlarged interference range by using cooperative communication technique.

To date, research work on CMAC protocols has mainly focused on the SDG of single links obtained by one or more helpers (H) in a fully-connected or two-hop network and on validating their effectiveness, such as improving network throughput and decreasing packet delay. However, in the larger scale wireless networks, numerous links need to reuse the same radio channel to communicate with each other due to the limits of wireless spectra, and the enlarged interference range (IR) of cooperative transmission links reduces the frequency reuse in the whole network. As shown in Figure 1, when a sender (S) sends a data packet to its recipient (D) and chooses H as the helper to increase the transmission rate of S to D, the packet transmission process between A and B is blocked, and therefore this reduces the total number of concurrent transmission links in the network, i.e., decreases the spatial frequency reuse gain (SFRG), which does not exist in direct transmissions. Thus, in large scale wireless networks, cooperation gain is determined by both SDG and SFRG. The tradeoff between these two conflicting factors should be carefully studied and the design of the CMAC protocol that can maximize the SFRG and SDG has become a research hotspot in multihop large scale wireless networks.

It is difficult to analyze the entire network throughput and the cooperation gain generated by cooperative communication due to the random positions of each packet transmission link and each helper in the large scale wireless networks. On one hand, whether a link needs node cooperation depends on the channel state (i.e., the instantaneous received signal-to-noise ratio, SNR) between S and D [3]. If the link needs node cooperation, cooperation gain is decided by the channel state between S-H and H-D. On the other hand, it is more difficult to analyze the reduced SFRG due to the random position of each helper in a multihop wireless network [8]. To date, research work has focused on improving SDG or SFRG, and very little research work exists evaluating the joint cooperation gain brought by SDG and SFRG in multihop wireless networks. The authors in a previous study [8] only considered the cooperation gain from the perspective of transmission reliability achieved by both SDG and SFRG, and ignored the helper selection time and packet transmission time.

In this paper, we investigate the impact of time in the process of helper selection and data packet transmissions on the network throughput and cooperation gain. Firstly, we propose a cooperative MAC protocol with optimal relay selection (ORS-CMAC) for multirate large scale wireless networks. Secondly, we analyze the interference range and single link throughput for direct transmission (DT) mode and cooperative transmission (CT) mode based on reasonable assumptions. According to the randomized exclusive region based (REX) scheduling scheme [9], the expected numbers of concurrent transmission links in DT and CT are derived as a function of the total number of links in the entire network and interference-free probability between any two links. Then, the network throughput, which is defined as the product of the throughput of single links and the number of concurrent transmission links, is derived. The cooperation gain of the whole network, which is defined as the ratio of the network throughput in CT to that in DT, is used to evaluate the MAC performance of the ORS-CMAC protocol.

The main contributions of this paper are as follows.

- To reduce the interference range introduced by the helpers, improve the spatial frequency reuse gain (SFRG), and thus improve the whole network throughput, we propose a cooperative MAC protocol with optimal relay selection, i.e., ORS-CMAC protocol. We design a helper selection scheme, including a cooperative priority differentiation (CPD) round and a contention for helper selection (CHS) round, to select a unique optimal helper promptly from all potential helpers with high probability and minimize the enlarged interference range introduced by the helper.
- We construct the interference range of a single link and derive the expression of SFRG (denoted by *ρ*) based on the REX scheduling scheme in a multirate network environment. The average packet transmission times of the direct transmission and cooperative transmission are analyzed, and then the SDG (denoted by *η*) is obtained. Based on these, the cooperation gain (denoted by *G*) of the entire network generated by cooperation transmissions, and the network throughputs in DT and CT mode are obtained.
- The effectiveness of the proposed mathematical model is verified by simulation results. The theoretical results and simulation results show that cooperative communication reduces the SFRG, but the increased SDG can compensate the decrease of SFRG, thus improving the whole network performance.

The rest of this paper is organized as follows. In Section 2, the related work is introduced. The network model adopted in this paper is described in Section 3. In Section 4, the proposed ORS-CMAC protocol is presented, including rapid helper selection strategy and packet transmission process. The interference ranges and network throughputs in DT and CT mode, and cooperation gain of the whole network are analyzed in Section 5. In Section 6, the theoretical results and simulation results are given. Finally, Section 7 concludes this paper.

## 2. Related Work

In general, two methods are used to improve network performance. One method aims to improve the throughput and transmission reliability of single links. Another method is to increase the spatial frequency reuse, and thereby increase the number of concurrent transmission links.

Recently, many CMAC protocols have been proposed for multihop wireless networks [10]. According to when the helper is chosen, they can be classified into proactive CMAC protocols [3,11,12], reactive CMAC protocols [13,14,15], and hybrid CMAC protocols [16,17,18,19].

In the proactive CMAC protocols, based on the received RTS/CTS packets, each node maintains a cooperative table (usually called CoopTable), which contains information about its neighboring nodes that can help it forward its data packet, and associated cooperative quality. Before the direct transmission, a sender should determine whether a helper can support a higher equivalent data rate to its recipient. If the helper can shorten the consumed time of its data packet transmission, it will choose one helper from its CoopTable, and insert the information about the helper into the related control packet. Otherwise, it will send its data packet to its recipient directly. Typical proactive CMAC protocols are CoopMAC [3], adaptive distributed cooperative relaying MAC (ADC-MAC) [11], and relay-enabled distributed coordination function (rDCF) [12]. In the CoopMAC protocol [3], if the direct transmission rate between a sender S and its recipient D is low, S will select one optimal helper from its CoopTable to forward its data packet, and thus shorten the entire transmission time of each data packet. In the ADC-MAC protocol [11], each node establishes its CoopTable through periodic heartbeats and uses the shortest path algorithm to select the optimal helper. The sender decides whether the cooperative transmission is adopted or not based on the SNR value in the helper-clear-to-send (HCTS) packet. If the SNR in the HCTS packet is larger than the given threshold, the sender will adopt the cooperative transmission. Otherwise, the sender will send its data packet directly to its recipient. In the rDCF protocol [12], each relay node periodically advertises its willing list to its one-hop neighbors, and based on this, the other nodes can set up their relay table. The sender designates one of the possible relay nodes in its relay RTS (RRTS1) packet and then the specified relay node sends another relay RTS (RRTS2) packet to the receiver. Based on the signal strength of RRTS1 and RRTS2 packets and exchanged information in these packets, the receiver obtains *r*_SD_, *r*_SH_, and *r*_HD_, and finally determines whether cooperation transmission is adopted. These kinds of cooperative MAC protocols determine the cooperative transmission and selects the helper based on the historical information, which may be out of date. Therefore, they are inefficient in dynamic change of network topology and link quality.

Typical reactive CMAC protocols are cooperative ad hoc MAC (CAH-MAC) protocol [13], network coding aware cooperative MAC (NCAC-MAC) protocol [14], and cooperative relaying MAC (CoRe-MAC) protocol [15]. In these protocols, the cooperative transmission is initiated only if the direct transmission fails. In the CAH-MAC protocol [13], if the direct transmission fails, the potential helpers that have overheard the data packet forward it only if the destination is reachable and there exist idle timeslots. In the NCAC-MAC protocol [14], the helper H will forward its received data packet and its own data packet using hybrid cooperative network coding (HCNC) if the signal-to-interference-plus-noise ratio (SINR) of the data packet received by the destination of the helper is larger than the given threshold. Otherwise, the helper will only forward the received data packet. The destination(s) of the sender and helper decode their data packets based on MIMO network coding (MIMO_NC) technique [20]. In the CoRe-MAC protocol [15], if the packet error ratio (PER) extracted from the request-to-send (RTS) packet is larger than the given threshold, the destination D will reply to the sender S with a cooperative-clear-to-send (CCTS) packet to start the cooperative transmission. If PER is lower than the given threshold, D will reply with a clear-to-send (CTS) packet, and the cooperative transmission is not triggered even if the direct transmission fails. All neighboring nodes should overhear the channel and receive all transmitted data packets for possible oncoming cooperation in the reactive CMAC protocol, and therefore this causes unnecessary energy wastage.

To solve the problems of the proactive CMAC and reactive CMAC protocols, the hybrid CMAC protocols allow the helper H to decide whether the cooperative transmission is needed based on the channel state of S-H and H-D obtained from the RTS/CTS handshake process. Typical hybrid CMAC protocols are CoopMAC-aggregation (CoopMACA) protocol [16], two-relay-based cooperative MAC (2rc-MAC) protocol [17], link-utility-based cooperative MAC (LC-MAC) protocol [18], and cross-layer cooperative MAC (CL-CMAC) protocol [19]. In the CoopMACA protocol [16], if the direct transmission rate between S and D is low, the helper selection process will be initiated. All potential high-data-rate helpers contend to be a unique helper through 3 round contentions to participate in the cooperative transmission. The 2rc-MAC [17] protocol chooses two potential optimal helpers to improve the throughput and transmission reliability. The potential helpers that support a higher equivalent cooperative transmission rate send their busy tones in their corresponding minislots of the relay response (RR) frame to declare their presence. Once the RR frame has finished, S chooses the two best helpers from all the potential helpers. The highest priority helper is used first. Once the cooperative transmission has failed, the other helper immediately forwards the data packet again. Besides considering the network throughput mentioned above, LC-MAC [18] uses the link-utility that considers both transmission rate and energy efficiency to select the optimal helper. CL-CMAC [19] proposes an optimal grouping strategy for efficient helper selection, and devises a greedy algorithm for MAC protocol refinement. In the case of multiple optimal helpers where two or more ready-to-help (RTH) packets collide, they resend their RTH packets in a randomly selected minislot from 𝐾 minislots. The hybrid CMAC protocols can obtain the instantaneous channel state of the network through RTS/CTS handshakes, and choose the best helper to participate in the node cooperation.

The CMAC protocols mentioned above only consider the case that one helper participates in each cooperative transmission. In the space-time code MAC (STiMAC) protocol [21], multiple helpers forward the data packet simultaneously through distributed space-time coding (DSTC), which can further improve the link throughput and transmission reliability. However, these helpers should coordinate closely and keep synchronization with each other, which increases the overhead and complexity. Meanwhile, the interference range (IR) of the protocol is enlarged because of the same data packet transmissions of multiple helpers. Therefore, these kinds of CMAC protocols cannot be efficiently used in large scale wireless networks.

Spatial frequency reuse is a critical factor to determine the network throughput in the large scale wireless networks [22]. In general, the tuning of the carrier sensing threshold, the transmission power control, the data rate adaptation, and the use of directional antennas are adopted to achieve spatial frequency reuse and concurrent transmissions [23]. Differing from previous research studies that use a fixed carrier sensing threshold, which can increase the interference range, the authors in one study [24] demonstrated that tuning the carrier sensing threshold is an effective manner to avoid interference and increase the network spatial frequency reuse in different network environments. Two optimal carrier sensing thresholds are derived, and an enhanced physical carrier sensing mechanism with tunable sensing threshold is adopted to improve network throughput by maximizing the potential of the spatial reuse. In another study [25], the authors studied the influence of different transmission ranges, receiving sensitivities, and multihop forwarding on the optimal physical carrier sensing range and demonstrated that the bandwidth distance product could be a good routing metric to maximize the spatial reuse ratio, and hence optimize the end-to-end performance of multihop flows when considering both multirate and carrier sensing ranges. In order to guarantee the quality-of-service (QoS) requirement in terms of delay, recent topology control algorithms jointly consider the interference constraint and delay constraint [26,27]. Based on the proposed delay models for a path and an intermediate node, the authors in a previous study [26] designed a cross-layer distributed topology control algorithm to control the transmission power of nodes to minimize the interference and satisfy the delay requirement. A previous study [27] proposed a centralized greedy strategy-based topology control algorithm to minimize the maximum interference while satisfying the delay constraint, a localized topology control algorithm to build a delay-constrained minimum spanning tree independently for each node to minimize the average interference, and a distributed delay-constrained Bellman-Ford topology control algorithm to find the optimal path with the average path interference. To reduce the interference among concurrent transmission links, some MAC protocols (e.g., IEEE 802.11p) employ a modulation scheme of orthogonal frequency division multiplexing (OFDM) without interference [28,29]. In a previous study [28], a concurrent transmission-based broadcast protocol is proposed to schedule the concurrent broadcast transmissions of the forwarders in the same divided segment in order to reduce the broadcast delay and increase the broadcast reliability. To meet the requirement of concurrent transmissions of OFDM signals, a time synchronization mechanism is proposed to make sure the maximum temporal displacement of the concurrent transmissions from adjacent forwarders in the same segment is satisfied in multihop broadcasting.

The above approaches on improving spatial frequency reuse only consider cases of direct transmissions. The cases become more complicated due to the enlarged interference range caused by the helper in the cooperative transmissions. The authors in a previous study [30] analyze the relationship among the interference range, the distance between S and D, and the position of the helper, and then construct a unit disk graph model of single links for the direct transmissions and cooperative transmissions. In order to mitigate the impact of the interference, the authors propose two channel allocation mechanisms, i.e., flexible channel partition and fixed channel partition, and their effectiveness is demonstrated by simulation results. However, due to the limited wireless channel resources, it is not possible to assign different channel resources to each communication node pair, especially in a large scale wireless network. Compared to the unit disk graph model, the elliptical region model given in a previous study [8] is more accurate. However, the authors study the influence of SDG and SFRG on the network performance only from the perspective of transmission reliability and ignore the effect of packet transmission time. In this paper, we evaluate the influence of SDG and SFRG on the network performance by taking into account the distribution of links and helper positions, and time-varying channel.

## 3. Network Model

### 3.1. Network Topology

We assume that the network consists of *N*_L_ senders and that each sender communicates with an associated recipient at a fixed communication distance *d*. All the links that are composed of senders and their associated recipients, i.e., sender-recipient (S-D) pairs, are uniformly distributed in a circular region with the radius of *R*_max_. To simplify the analysis, we assume that all the links have packets to transmit all the time [31]. The helpers H are uniformly distributed in the same region with node density λ_H_. Therefore, the probability that a region with an area of *A*_0_ has at least one helper is

(1) $$P\{N_{A_{0}}\geq 1\}=P\{N_{A_{0}}\neq 0\}=1-P\{N_{A_{0}}=0\}=1-{\left(1-A_{0}/A_{T}\right)}^{\lambda_{H}A_{T}}$$

where $A_{T}=\pi R_{\max}^{2}$ is the area of the circular region, and λ_H_*A*_T_ is the total number of helpers in this region. In the network, we assume that each helper is ready to participate in the cooperative transmissions all the time.

### 3.2. Packet Transmission Model

**Figure 2:**
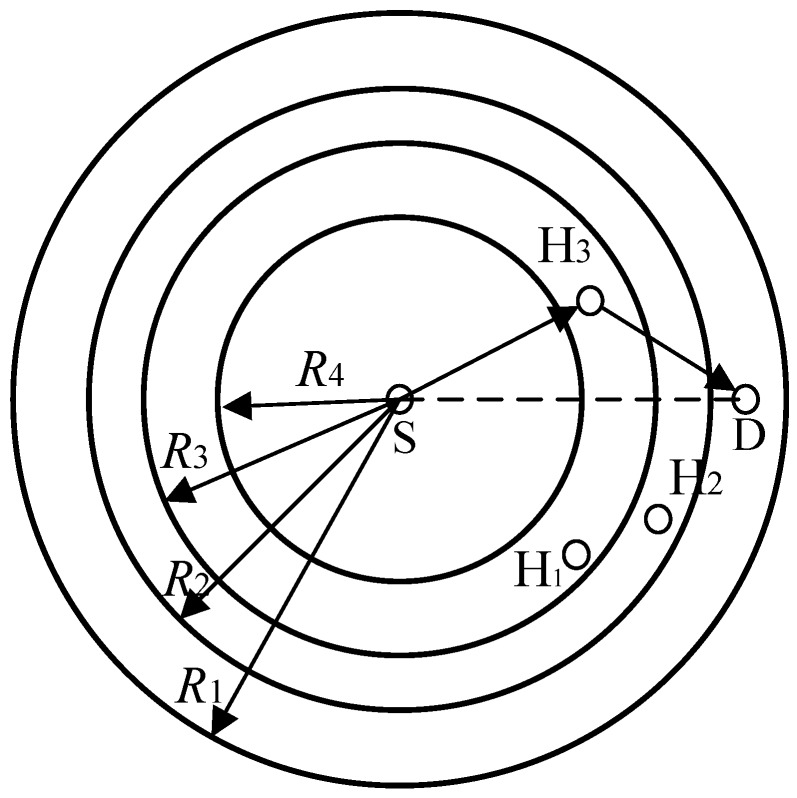
Transmission ranges for the links with different data rates.

All nodes in the network transmit their packets at a fixed transmission power, and each node is equipped with a half-duplex transceiver with an omnidirectional antenna. In this paper, we adopt the IEEE 802.11b with four different transmission rates *r*_1_, *r*_2_, *r*_3_ and *r*_4_ (i.e., 1 Mbps, 2 Mbps, 5.5 Mbps and 11 Mbps), whose corresponding transmission ranges are *R*_1_, *R*_2_, *R*_3_, and *R*_4_, respectively. As shown in Figure 2, with the sender as the center, a series of circles are formed by these four different transmission ranges. Control packets and the headers of PHY and MAC layers are transmitted at the basic rate *R*_b_, and *R*_b_ = *r*_1_.

For the data packet transmission, if the direct transmission rate (denoted by *r*_SD_) is *r*_1_ or *r*_2_, the cooperative transmission will be triggered, and a helper with the maximum cooperation rate will be selected to forward the data packet for the sender (as H_3_ in Figure 2). Otherwise, S will send its data packet to D directly.

For simplicity, we assume that the positions of S, H, and D and the channel state are fixed during each data packet transmission process. The data packet is dropped as long as packet collisions exist during its transmission. That is to say, each link loses its transmission reliability with the existence of other links within its transmission region.

## 4. Cooperative MAC Protocol with Optimal Relay Selection

In the cooperative MAC protocol with optimal relay selection (ORS-CMAC), the helpers with higher equivalent cooperative transmission rates help the links with lower direct transmission rates. This protocol can be classified into three phases, i.e., reservation phase, helper selection phase, and data packet transmission phase.

### 4.1. Reservation Phase

**Figure 3:**
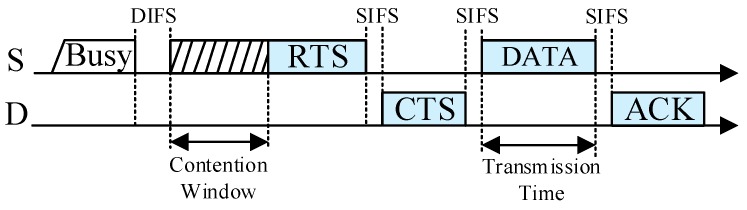
Direct transmission.

Once a sender S needs to transmit its data packet to its recipient D, it uses the distributed coordination function (DCF) to access the channel. The sender S that accesses the channel successfully sends its RTS packet to its recipient D and D replies with a CTS packet if it receives the RTS packet correctly. Through the RTS/CTS handshake, both S and D obtain the maximum direct transmission rate. The neighboring nodes that overhear and decode the RTS/CTS packets successfully become the potential helpers, and can obtain the maximum transmission rates between itself and S, and between itself and D, denoted by *r*_SH_ and *r*_HD_, respectively. If *r*_SH_ and *r*_HD_ satisfy *r*_C_ = 1/(1/*r*_SH_ + 1/*r*_HD_) < *r*_SD_, i.e., the equivalent cooperative transmission rate *r*_C_ is lower than the direct transmission rate *r*_SD_, S will send its data packet to D directly, as shown in Figure 3. Otherwise, S will send its data packet to D through the cooperative transmission.

### 4.2. Helper Selection Phase

To minimize the enlarged interference introduced by the helper, ORS-CMAC only selects one optimal helper with the highest equivalent cooperative transmission rate to participate in each cooperative transmission. The process of selecting the helper is divided into the cooperative priority differentiation (CPD) round and contention for helper selection (CHS) round.

#### 4.2.1. Cooperative Priority Differentiation (CPD) Round

In order to maximize the cooperative diversity gain, we choose the helper with the highest equivalent cooperative transmission rate. The neighboring node will be one of the potential helpers, if it satisfies

(2) $$T_{CPD}+T_{CHS}+3SIFS+T_{HTS}+T_{HEADER}+L_{PKT}/r_{C}<L_{PKT}/r_{SD}$$

where *T*_CPD_ and *T*_CHS_ are the times spent in the processes of CPD and CHS, SIFS is the short interframe space, *T*_HEADER_ = *L*_HEADER_/*R*_b_ is the total transmission time of the MAC header and PHY header, *L*_PKT_ is the length of a data packet, and *r*_SD_ is the direct transmission rate between S and D. The helper that wins in the two contention rounds sends a helper-to-send (HTS) packet to S and D, and the corresponding time is *T*_HTS_ = *L*_HTS_/*R*_b_. Here, *L*_HTS_ represents the length of the HTS packet. The equivalent cooperative transmission rate *r*_C_ can be rewritten as

(3) $$r_{C}=\frac{r_{SH}r_{HD}}{r_{SH}+r_{HD}}$$

where *r*_SH_ and *r*_HD_ are the transmission rates between the sender S and the helper, and between the helper and the recipient D, respectively.

In CPD, all potential helpers transmit their busy tones in different minislots to distinguish the cooperation priority. We denote *G*_R_ as the cooperation gain, which can be written as *G*_R_ = *r*_c_*/r*_SD_. All potential helpers determine their cooperative priority levels according to *G*_R_. The cooperative priorities from high to low are designated as *G*_R1_, *G*_R2_, …, and *G*_RP_, respectively. Here, *P* is the total number of cooperative priorities. CPD includes at most *P* minislots for distinguishing different cooperative priorities. A potential helper determines its cooperative priority *G*_Ri_ (*i* = 1, 2, …, *P*) based on its *r*_C_ and *r*_SD_, and if it does not sense the busy tone before *i*-th minislot, it transmits its busy tone in its predefined *i*-th minislot.

The relationship between data rate combination (*r*_SH_, *r*_HD_), priority level, cooperative transmission rate, and corresponding minislot can be seen from Table 1 when *r*_SD_ = 1 Mbps. When *r*_SD_ = 2 Mbps, the maximum number of minislots in CPD is three and the corresponding minislots of (*r*_4_, *r*_4_), (*r*_3_, *r*_4_) and (*r*_4_, *r*_3_), and (*r*_3_, *r*_3_) are 1, 2, and 3, respectively. The time duration of each minislot is τ.

**Figure 4:**
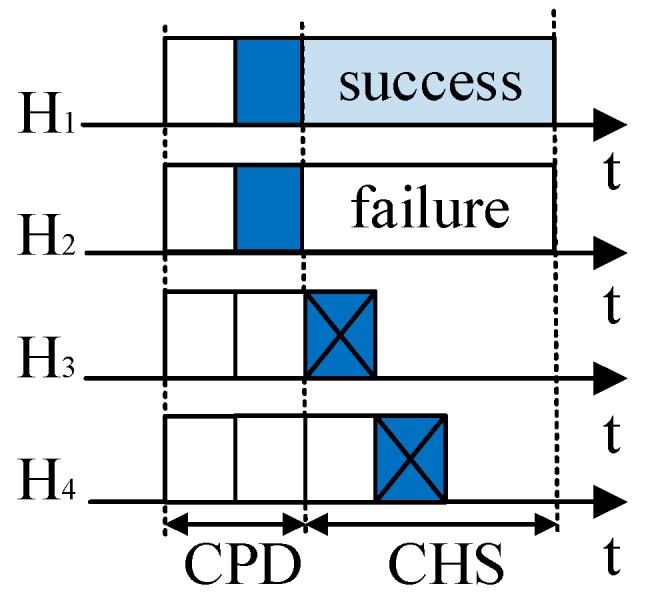
Cooperative transmission with only one helper wins in the helper selection phase.

**Figure 5:**
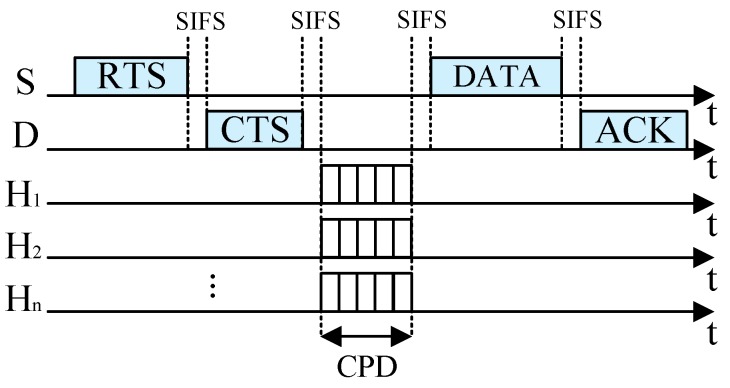
Direct transmission for lack of helpers in CPD.

In order to decrease the time spent in the priority differentiation round, RRS-CMAC [32] defines a virtual ID consisting of *n*_B_ ($n_{B}=\left\lceil \log_{2}P\right\rceil$, where $\left\lceil \log_{2}P\right\rceil$ is the smallest integer equal to or greater than $\log_{2}P$) binary digits to represent rate priority in rate differentiation phases. For example, RRS-CMAC only needs three minislots to distinguish five priorities, but ORS-CMAC needs five minislots. However, the binary digits of (*r*_4_, *r*_4_) and (*r*_3_, *r*_3_) are 111 and 101 according to Table 2 in a previous study [32]. That is to say, all potential helpers that support (*r*_4_, *r*_4_) and (*r*_3_, *r*_3_) send their busy tones in the first minislot. According to Section 5 of this paper, the helpers that support (*r*_3_, *r*_3_) have a larger interference range. Therefore, RRS-CMAC cannot be applied to large scale wireless networks directly. To decrease the unnecessary interference, in ORS-CMAC, the potential helper with cooperation gain G_Ri_ (*i* = 1, 2, 3, …, *P*) sends its busy tone in its predefined *i*-th minislot and senses the channel before the *i*-th minislot. If there are busy tones in the channel before the *i*-th minislot, i.e., other helpers send their busy tones earlier than it (e.g., H_3_ and H_4_ in Figure 4), the CPD process ends immediately. Otherwise, the helper sends its busy tone in its predefined *i*-th minislot (e.g., H_1_ and H_2_ in Figure 4), and the CPD process ends. All potential helpers that send their busy tones in the CPD win the contention and enter the next stage of contention. They should not be interfered with by other transmission links and they enter into the CHS round. If there is no busy tone in the whole process of CPD, it will indicate that there is no available helper, and S will send its data packet to D directly, as Figure 5 shows.

#### 4.2.2. Contention for Helper Selection Round

The helpers that win in the CPD process have the highest priority and the largest cooperative transmission rate. However, there may be more than one such helper. In order to select the unique optimal helper rapidly, the *k* round contention resolution (*k*-CR) [33] is adopted in the CHS process.

**Figure 6:**
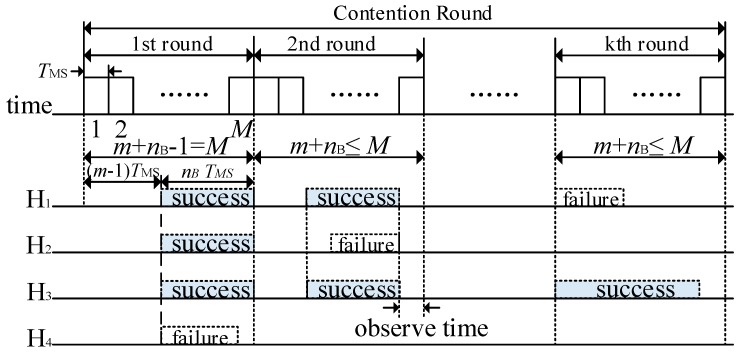
Timing structure of *k*-CR process.

There are *k* rounds of contention with at most *M* contention minislots in each round in *k*-CR, as shown in Figure 6. The potential helpers that win in the CPD randomly select the *m*-th (1 ≤ *m* ≤ *M*) minislot to start sending a busy tone with a length of *n*_B_ (1 ≤ *n*_B_ ≤ *M*, and *m* + *n*_B_ − 1 ≤ *M*) minislots. Each potential helper senses the channel before sending its own busy tone. If there is a busy tone in the channel before its busy tone transmission, it will withdraw from the contention (e.g., H_2_ in the second round in Figure 6). Otherwise, it will send its predefined busy tone. Once a potential helper finishes its own busy tone transmission, it will observe the channel for one minislot if there are remaining minislots in the current round (i.e., *m* + *n*_B_ ≤ *M*). If it senses other busy tones, it will abandon the remaining contentions (e.g., H_4_ in the first round). Otherwise, it will participate in the next round of contention (e.g., H_1_ and H_3_ in the second round in Figure 6). However, if there is no minislot left (i.e., *m* + *n*_B_ − 1 = *M*), the potential helpers with the earliest busy tone transmission will continue to the next round directly (i.e., H_1_, H_2_, and H_3_ in the first round in Figure 6). Therefore, the potential helpers with the earliest busy tone transmissions and the longest busy tone duration win in each round and continue to the next round of contention until the end of *k*-CR process.

### 4.3. Data Packet Transmission Phase

According to the result of the CHS process, the data packet transmission process can be classified into two cases.

**Figure 7:**
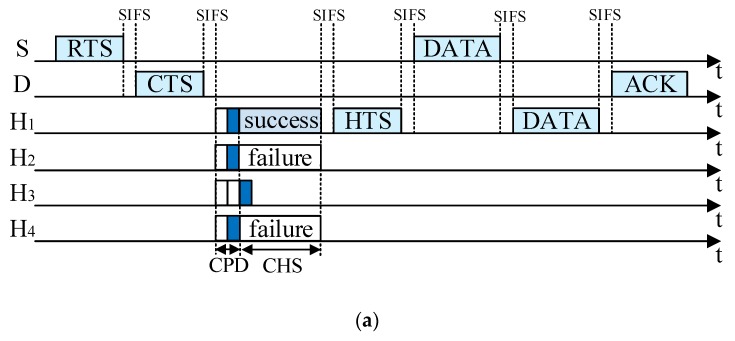

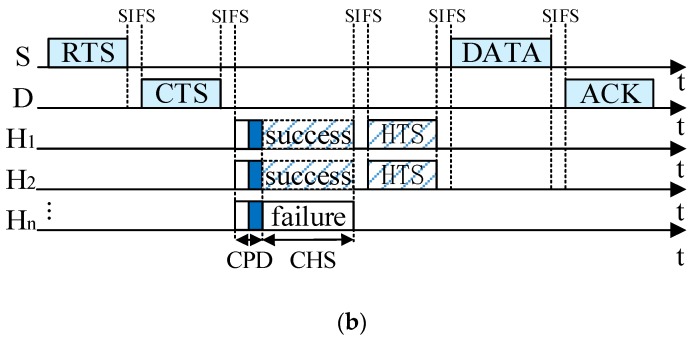
Timing structure of cooperative transmission: (**a**) Only one helper wins in the helper selection phase; (**b**) multiple potential helpers win in the helper selection phase.

(1) If only one potential helper wins in the CHS, it will send a HTS packet to declare itself as the helper using basic rate *R*_b_ with *r*_SH_ and *r*_HD_ in it. Then, S sends its data packet to H with the data rate *r*_SH_, and H forwards the received data packet to D with the data rate *r*_HD_. When D successfully decodes the data packet forwarded by H, it replies S with an acknowledgment (ACK) packet. In Figure 7a, the helpers that support (*r*_3_, *r*_4_) or (*r*_4_, *r*_3_) have the highest priority and they send busy tones in the second minislot in CPD. Thus, the length of CPD is only two minislots. In the CHS process, only H_1_ wins the contention and S sends its data packet to H_1_ using *r*_SH_ (*r*_SH_ = 5.5 Mbps or 11 Mbps). Then, H_1_ forwards the data packet to D with *r*_HD_ (*r*_HD_ = 11 Mbps or 5.5 Mbps). Finally, D replies S with an ACK packet.

(2) If there are at least two helpers winning in the CHS process, they send their HTS packets simultaneously, which causes packet collisions at S and D. Thus, S sends its data packet to D directly. As shown in Figure 7b, both H_1_ and H_2_ win in the CHS process and they send their HTS packets at the same time. Therefore, S sends its data packet to D directly. After D receives and decodes the data packet successfully, it replies to S with an ACK packet.

## 5. Performance Analysis

In this section we analyze the network throughput and cooperation gain of ORS-CMAC from the viewpoint of the whole network. For simplicity, we ignore access collisions among all senders and recipients, and give their upper bounds. We define the network throughput as the multiplication of the single link throughput and the number of concurrent transmission links. Let *S*_D_ and *S*_C_ be the network throughputs in DT and CT mode, respectively. They can be written as

(4) $$S_{D}=N_{D}L_{PKT}/\bar{T_{D}}$$

(5) $$S_{C}=N_{C}L_{PKT}/\bar{T_{C}}$$

where $\bar{T_{D}}$ and $\bar{T_{C}}$ are the average packet transmission times in DT and CT, and *N*_D_ and *N*_C_ are the corresponding numbers of concurrent transmission links in the whole network, respectively.

In DT mode, the number of concurrent transmission links (i.e., *N*_D_) is determined by the combination interference range of S and D. However, *N*_C_ is further affected by the interference range of the helper H. That is to say, the helpers with different positions have different influences on the number of concurrent transmission links in CT mode. It is obvious that the number of concurrent transmission links in CT mode is less than or equal to the number of concurrent transmission links in DT mode, i.e., *N*_C_ ≤ *N*_D_. Therefore, cooperative transmissions can improve SDG at the expense of reducing SFRG. To evaluate the cooperation gain generated by CT, we define it as the ratio of network throughput in CT mode to that in DT mode, which can be written as

(6) $$G=S_{C}/S_{D}=\frac{N_{C}}{N_{D}}\cdot \frac{\bar{T_{D}}}{\bar{T_{C}}}=\rho \cdot \eta$$

where *ρ* = *N*_C_/*N*_D_ represents the spatial frequency reuse gain and $\eta =\bar{T_{D}}/\bar{T_{C}}$ is the spatial diversity gain of cooperative transmissions.

### 5.1. Spatial Frequency Reuse Gain

In this section, we construct the interference model of the direct transmission link and cooperative transmission link, respectively. The number of concurrent transmission links in DT and CT can be obtained based on the REX scheduling scheme [9], and then the spatial frequency reuse gain is derived.

With reference to a previous study [8], we denote *P*(*k*, *n*) as the probability that *k* S-D pairs can transmit concurrently when there are *n* S-D pairs in a large scale wireless network. Let *q* be the interference-free probability between any two S-D pairs. In the cases that there are *k* S-D pairs that can transmit concurrently for *n* S-D pairs, these can be divided into the following two cases:

(1) For *n*-1 S-D pairs, there are *k*-1 S-D pairs that can be scheduled concurrently and the *n*-th S-D pair is interference-free with the existed *k*-1 S-D pairs.
(2) There are *k* S-D pairs that can perform concurrent packet transmissions for *n*-1 S-D pairs and the *n*-th S-D pair is interfered with by at least one of the scheduled *n*-1 S-D pairs.

Therefore, *P*(*k*, *n*) can be written as follows

(7) $$P(k,n)=P(k-1,\;n-1)q^{k-1}+P(k,n-1)(1-q^{k})$$

Obviously, *P*(1, 1) = 1, *P*(1, 2) = 1−*q*, and *P*(2, 2) = *q*. For any 1 ≤ *k* ≤ *n*, *P*(*k*, *n*) can be calculated through an iterative approach using Equation (7). Denote *N*_0_ as the expected number of concurrent transmission links and it can be written as $N_{0}=\sum_{k=1}^{N_{L}}kP\left(k,N_{L}\right)$ on the condition that the total number of links in the network is *N*_L_. To obtain *N*_0_, the interference-free probability *q* needs to be computed at first, which is determined by the IR of the direct transmission link or cooperative transmission link. The IR of a direct transmission link is determined by the combination IRs of S and D. However, the IR of a cooperative transmission link is further determined by the position of the helper. The IRs of both the direct transmission link and cooperative transmission link can be approximated by the elliptical region [8]. Although the elliptical region model of interference is conservative, it is more accurate than the unit disk model proposed in [30]. Therefore, we adopt the elliptical region model to calculate *q* in this paper.

#### 5.1.1. Direct Transmission

**Figure 8:**
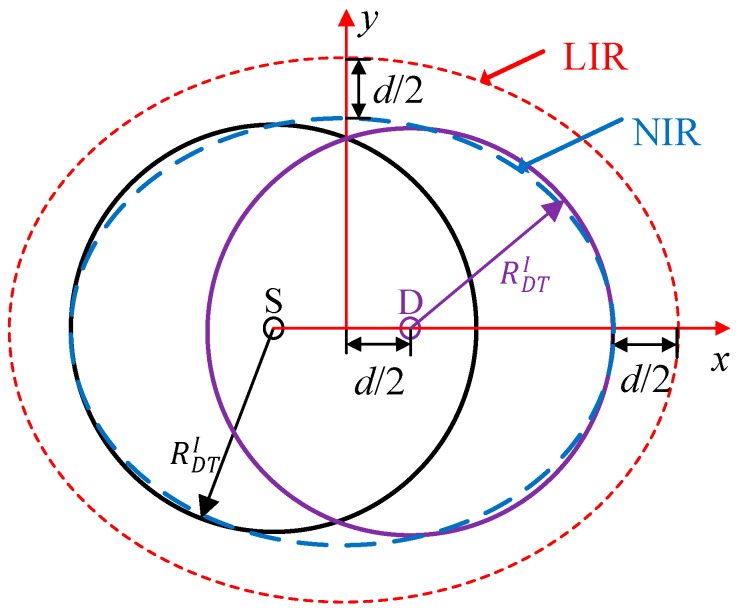
Link interference region and node interference region of direct transmission links.

As shown in Figure 8, the IR of the direct transmission S_i_−D_i_ pair is modeled as an elliptical region centered at the midpoint of S_i_ and D_i_ and is referred to as the node interference region (NIR_i_). Any other S-D pairs (e.g., S_j_-D_j_ (*j* ≠ *i*)) that can be scheduled concurrently should be located outside of NIR_i_ to avoid interfering with S_i_ and D_i_. As described in Section 3, the distance *d* between S and D is fixed, and thus the positions of S and D are not independent. To simplify the analysis, the link interference region (LIR) is introduced to describe the interference relationship among different links, which is also an elliptical region centered at the midpoint but larger than NIR [8]. As shown in Figure 8, the semi-major axis and the semi-minor axis of the LIR can be written as

(8) $$L_{DT}^{L}=R_{DT}^{I}+d$$

(9) $$L_{DT}^{S}=R_{DT}^{I}+\frac{d}{2},$$

where, $L_{DT}^{L}$ and $L_{DT}^{S}$ are the semi-major axis and semi-minor axis of LIR, respectively, and they are increased by *d*/2 compared to NIR, as shown in Figure 8. Further, $R_{DT}^{I}=(1+\alpha )R_{T}$ [34] is the interference radius of each node, and *R*_T_ = *R*_1_ is the transmission radius of the basic transmission rate. Therefore, the interference range of DT can be expressed as $A_{D}=\pi L_{DT}^{L}L_{DT}^{S}$. For any two direct transmission links L_i_ and L_j_ (*j* ≠ *i*), if the center of L_j_ is outside of the interference range of L_i_, they are interference-free. Therefore, the probability that any two direct transmission links are interference-free can be written as *q*_D_ = 1−*A*_D_/*A*_T_.

#### 5.1.2. Cooperative Transmission

Let A_ij_ be the area that the equivalent cooperative transmission rate of the helper in this area is *r*_C_ (*r*_C_ = *r*_i_*r*_j_/(*r*_i_ + *r*_j_), *r*_C_ > *r*_SD_), i.e., the helpers in this area can support the two-hop rate (*r*_SH_, *r*_HD_) between S and H, and H and D with (*r*_i_, *r*_j_) or (*r*_j_, *r*_i_). We suppose the direct transmission rate is *r*_SD_ = 1 Mbps in this paper. The same method can be applied to the case where *r*_SD_ = 2 Mbps. Table 2 shows the relationship between data rate combination (*r*_SH_, *r*_HD_) and its corresponding area A_ij_ as shown in Figure 11.

**Figure 9:**
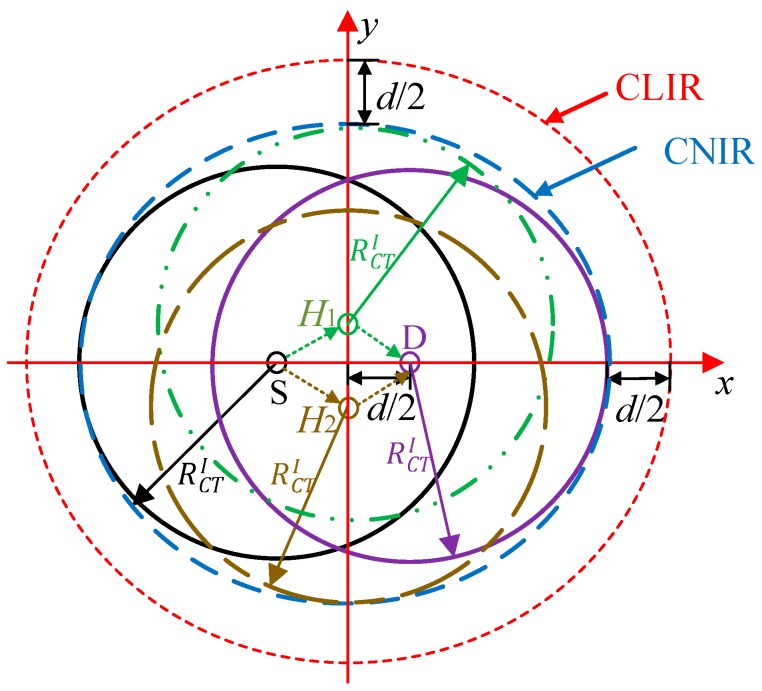
Link interference region and node interference region of cooperative transmission links.

**Figure 10:**
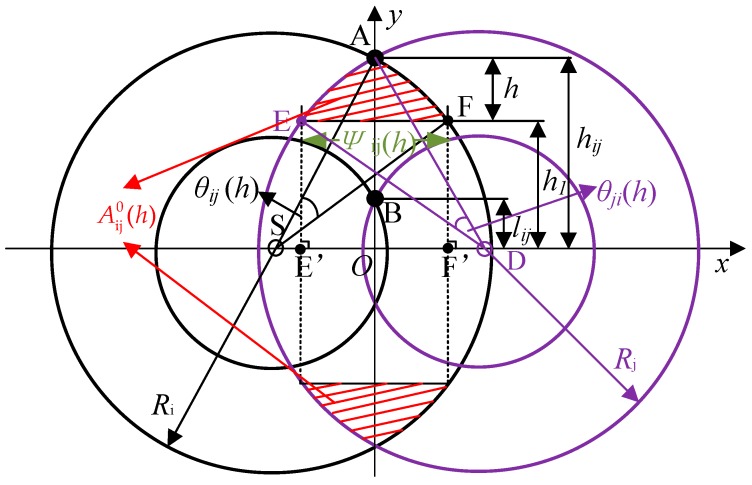
Overlapping area of the helpers with data rate combination (*r*_i_, *r*_j_) support.

**Figure 11:**
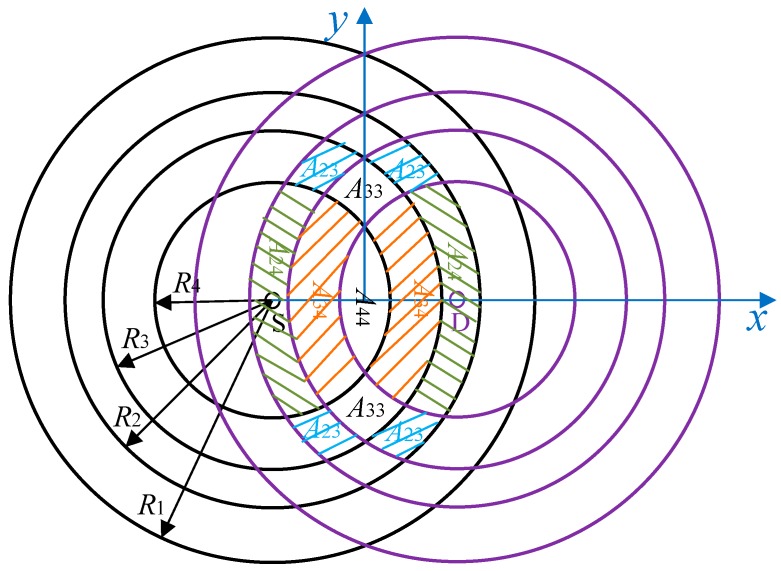
Overlapping area of a sender S and its recepient D, and helper regions.

Compared to direct transmission links, the IR of cooperative transmission links is further determined by the relative location of the furthermost helper. As shown in Figure 9, the larger the absolute value of Y coordinate the helper has, the larger the IR of the cooperative transmission links is. We denote *h*_ij_ and *l*_ij_ as the largest and smallest Y coordinates of A_ij_ above the link, respectively. For example, as shown in Figure 10, A and B are the top point and bottom point of A_ij_, respectively. From [35], *h*_ij_ can be written as $h_{ij}=\sqrt{2R_{i}^{2}R_{j}^{2}+2\left(R_{i}^{2}+R_{j}^{2}\right)d^{2}-\left(R_{i}^{4}+R_{j}^{4}\right)-d^{4}}/2d$. It can be seen from Figure 11 that *l*_44_ = *l*_34_ = *l*_24_ = 0, *l*_33_ = *h*_44_, *l*_23_ = *h*_34_. It is obvious that *h*_ij_ = *h*_ji_ and *l*_ij_ = *l*_ji_, due to the symmetry of A_ij_. We denote *h*_1_ as the Y coordinate’s absolute value of the helpers in A_ij_. Let *H*_ij_ be the expected largest value of *h*_1_ in A_ij_, which can be derived from Equations (32) and (33) in a previous study [8] as follows

(10) $$H_{ij}=h_{ij}-\int_{0}^{h_{ij}-l_{ij}}(1-\frac{2A_{ij}^{A}(h)}{A_{T}}{)}^{\lambda_{H}A_{T}}dh$$

where $A_{ij}^{A}(h)$ is the size of the area of A_ij_ with *y* ≥ *h*_ij_ − *h*, and *h* is the difference between the highest Y coordinate of the corresponding area *h*_ij_ and the possible largest Y coordinate of the helper *h*_1_ in this region. Then, $A_{ij}^{A}(h)$ can be written as

(11) $$A_{44}^{A}(h)=\left\{ \begin{array}{ll} A_{{}_{44}}^{0}(h)/2, & d\leq 2R_{4}\\ 0, & 2R_{4}<d\leq R_{1} \end{array}\right.$$

(12) $$A_{34}^{A}(h)=\left\{ \begin{array}{ll} A_{34}^{0}(h), & h\in [0,h_{34}-h_{44}]\\ A_{34}^{0}(h)-2A_{44}^{A}(h+h_{44}-h_{44}), & h\in (h_{34}-h_{44},h_{34}] \end{array}\right.$$

(13) $$A_{33}^{A}(h)=\left\{ \begin{array}{ll} A_{33}^{0}(h)/2, & h\in [0,h_{33}-h_{34}]\\ A_{33}^{0}(h)/2-A_{34}^{A}(h+h_{44}-h_{34}), & h\in (h_{33}-h_{34},h_{33}-h_{44}] \end{array}\right.$$

(14) $$A_{24}^{A}(h)=\left\{ \begin{array}{ll} A_{24}^{0}(h), & h\in [0,h_{24}-h_{34}]\\ A_{24}^{0}(h)-A_{34}^{0}(h+h_{34}-h_{24}), & h\in (h_{24}-h_{34},h_{24}] \end{array}\right.$$

(15) $$A_{23}^{A}(h)=\left\{ \begin{array}{ll} A_{23}^{0}(h), & h\in [0,h_{23}-h_{33}]\\ A_{23}^{0}(h)-A_{33}^{A}(h+h_{33}-h_{23}), & h\in (h_{23}-h_{33},h_{23}-h_{24}]\\ A_{23}^{0}(h)-A_{33}^{A}(h+h_{33}-h_{23})-A_{24}^{A}(h+h_{24}-h_{23}), & h\in (h_{23}-h_{24},h_{23}-h_{34}] \end{array}\right.$$

where $A_{ij}^{0}(h)$ is the size of the area formed by *y* ≥ *h*_ij_ − *h*, and two circles with the centers at S and D, and the radiuses *R*_i_ and *R*_j_, respectively, as the red shadow area shown in Figure 10. Equations (11)–(15) mean that $A_{ij}^{A}(h)$ is the size of the region with Y coordinate *y* larger than the helpers’ largest Y coordinate *h*_ij_-*h* in the area A_ij_ for the same cooperative priority with the transmission rate (*r*_i_, *r*_j_) or (*r*_j_, *r*_i_), which is also equal to that of the intersection area of these two cooperative transmission circles above the *X*-axis $A_{ij}^{0}(h)$, subtracting the size of the overlapping area with all higher cooperative priorities when the overlapping area exists. Note that for different pair (*i*, *j*), the numbers of subareas of A_ij_ are different, as shown in Figure 11. The size of the red shadow area $A_{ij}^{0}(h)$ in Figure 10 is composed of three parts, i.e., the double area of the triangle AEF (where E and F are the intersection points of the line *y* = *h_ij_* − *h* and the two circles with radiuses *R*_i_ and *R*_j_), the double areas of the differences between two triangles ASF and ADE, and their associated fans. Therefore, it can be expressed as

(16) $$A_{ij}^{0}(h)=\psi_{ij}(h)h+R_{i}^{2}[(\theta_{ij}(h)-\sin\theta_{ij}(h)]+R_{j}^{2}[(\theta_{ji}(h)-\sin\theta_{ji}(h)]$$

where

(17) $$\psi_{\mathrm{ij}}(h)=\sqrt{R_{i}^{2}-{\left(h_{ij}-h\right)}^{2}}+\sqrt{R_{j}^{2}-{\left(h_{ji}-h\right)}^{2}}-d$$

(18) $$\theta_{ij}(h)=\arcsin(\frac{h_{ij}}{R_{i}})-\arcsin(\frac{h_{ij}-h}{R_{i}})$$

(19) $$\theta_{ji}(h)=\arcsin(\frac{h_{ji}}{R_{j}})-\arcsin(\frac{h_{ji}-h}{R_{j}})$$

where $\psi_{\mathrm{ij}}(h)$ is the distance between E and F, as shown in Figure 10. E’ and F’ are the projections of E and F on the *X*-axis, respectively. Therefore, we have $\psi_{\mathrm{ij}}(h)={SF}^{\prime }+{DE}^{\prime }-d$, as shown in Equation (17). Further, *θ*_ij_(*h*) is the angle formed by A and F with the vertex at S, and *θ*_ji_(*h*) is the angle formed by A and E with the vertex at D. From Figure 10, we have *θ_ij_*(*h*) = ∠*ASF* = ∠*ASO* − ∠*FSF*’ = arcsin(*OA/SA*) − arcsin(*F*’*F*/*SF*), as shown in Equation (18), and *θ_ji_*(*h*) = ∠*ADE* = ∠*ADO-*∠*EDE*’ = arcsin(*OA/DA*) − arcsin(*E’E/DE*), as shown in Equation (19).

As shown in Figure 9, we approximate the IR of cooperative transmission links as an elliptical region using the same method as direct transmission links. Therefore, the semi-major axis and semi-minor axis can be written as

(20) $$\bar{L_{CT}^{L}}=R_{CT}^{I}+d$$

(21) $$\bar{L_{CT}^{S}}=R_{CT}^{I}+d/2+E[H(d)]$$

where $R_{CT}^{I}$ is the interference radius in CT, and $R_{CT}^{I}=(1+\beta )R_{T}$. Let Ω_1_ represent the entire space of all A_ij_ when *r*_SD_ = 1 Mbps; *E*[H(*d*)] is the average largest Y coordinate of the helpers in different areas *H*_ij_ and can be written as

(22) $$E[H(d)]=\sum_{A_{ij}\in \Omega_{1}}H_{ij}P_{ij}$$

where *P*_ij_ is the probability that the optimal helper is in A_ij_ and can be written as

(23) $$P_{44}=P_{{}_{44}}^{0}$$

(24) $$P_{34}=P_{{}_{34}}^{0}(1-P_{44}^{0})$$

(25) $$P_{33}=P_{33}^{0}(1-P_{34}^{0})(1-P_{44}^{0})$$

(26) $$P_{24}=P_{24}^{0}(1-P_{33}^{0})(1-P_{34}^{0})(1-P_{44}^{0})$$

(27) $$P_{23}=P_{{}_{23}}^{0}(1-P_{{}_{24}}^{0})(1-P_{33}^{0})(1-P_{34}^{0})(1-P_{44}^{0})$$

where $P_{ij}^{0}$ is the probability that there is at least one helper in A_ij_, and (1 − $P_{ij}^{0}$) is the probability that there is no helper in A_ij_. $P_{ij}^{0}$ can be computed as follows by using Equation (1)

(28) $$P_{ij}^{0}=1-{\left(1-\frac{2A_{ij}^{A}(h_{ij}-l_{ij})}{A_{T}}\right)}^{\lambda_{H}A_{T}}$$

For any two cooperative transmission links L_i_ and L_j_ (*j*≠*i*), if the center of L_i_ is outside of the region of L_j_, their transmissions can be scheduled concurrently. The average interference range of CT link is $A_{C}=\pi \bar{L_{CT}^{L}L_{CT}^{S}}$. Therefore, the average probability of any two CT links that are interference-free can be written as $q_{C}=1-A_{C}/A_{T}$.

The value of *P*(*k*, *n*) can be obtained by substituting *q*_D_ and *q*_C_ into Equation (7). Then, the expected number of concurrent transmission links *N*_0_ and the spatial frequency reuse gain *ρ* can also be obtained.

### 5.2. Spatial Diversity Gain

For simplicity, we ignore the access collision case in the RTS/CTS handshakes. That is to say, we suppose that all links that are interference-free can always access the channel successfully. In this section, we evaluate the network performance from the viewpoint of the time spent on data packet transmission and the helper selection process.

As discussed in Section 4, S sends its data packet to D directly when *r*_SD_ = 11 Mbps or *r*_SD_ = 5.5 Mbps. Thus, the direct transmission time is $\bar{T_{D}}=T_{\mathrm{RTS}}+T_{\mathrm{CTS}}+3\mathrm{SIFS}+T_{\mathrm{HEADER}}+L_{\mathrm{PKT}}/r_{\mathrm{SD}}+T_{ACK}$. If the direct transmission rate *r*_SD_ is equal to *r*_1_ or *r*_2_, the cooperative transmission is adopted and the corresponding transmission time can be written as $\bar{T_{C}}=\bar{T_{CPD}}+\bar{T_{CHS}}+\bar{T_{PKT}}$. Here, $\bar{T_{CPD}}$, a and $\bar{T_{PKT}}$ are the average times used in CPD, CHS, and data packet transmission, respectively. That is to say, $\bar{T_{CPD}}$ is the expected time consumed in CPD by selecting the helpers with different priorities or using the direct transmission if no suitable helpers can participate in the cooperative transmission, $\bar{T_{CHS}}$ is the expected time of choosing the optimal helpers in each area A_ij_ in the process of CHS, and $\bar{T_{PKT}}$ is the average transmission time of each data packet by using possible (*r*_i_, *r*_j_) or (*r*_j_, *r*_i_) types of cooperative transmission when there is only one helper selected after CHS, or using direct transmission when more than one helper is selected after CHS or no helpers exist in all possible cooperative regions. These can be written as

(29) $$\bar{T_{CPD}}=\tau P_{44}+2\tau P_{34}+3\tau P_{33}+4\tau P_{24}+5\tau P_{23}+5\tau P_{f}$$

(30) $$\bar{T_{CHS}}=\sum_{A_{ij}\in \Omega_{1}}\tau P_{ij}L_{ij}$$

(31) $$\bar{T_{PKT}}=\sum_{A_{ij}\in \Omega_{1}}P_{ij}[P_{S-ij}T_{S-ij}+(1-P_{S-ij})T_{f-ij}]+P_{f}(SIFS+T_{D})$$

where τ is thet time duration of each minislot, and *P_f_* is the probability that there is no available helper and $P_{f}=\left(1-P_{23}^{0}\right)\left(1-P_{24}^{0}\right)\left(1-P_{33}^{0}\right)\left(1-P_{34}^{0}\right)\left(1-P_{44}^{0}\right)=\left[1-P_{23}^{0}(d)\right]P_{23}(d)/P_{23}^{0}(d)$. Ω_1_ represents the entire space of all A_ij_, as shown in Figure 11. *P*_S-ij_ is the probability of choosing only one optimal helper in the area A_ij_ successfully and *L*_ij_ is the average number of minislots used in the CHS to select the helper in this area. Further, *L*_ij_, and *P*_S-ij_ can be calculated from Equations (1) and (2) in a previous study [36]. *T_S_*_-ij_ is the average transmission time of a data packet using a helper in A_ij_ and it can be expressed as *T*_S-ij_ = *T*_RTS_ + *T*_CTS_ + 6SIFS + *T*_HTS_ + 2*T*_HEADER_ + *T*_ACK_ + *L*_PKT_/*r*_Ci_. *T_f_*_-*ij*_ is the average transmission time of a data packet if at least two helpers simultaneously send their HTS packets, and *T_f_*_-*ij*_ = *T*_RTS_ + *T*_CTS_ + 5SIFS + *T*_HTS_ + *T*_HEADER_ + *T*_ACK_ + *L*_PKT_/*r*_SD_.

Based on the above analysis, we can get the average transmission times in DT and CT, respectively. Therefore, spatial diversity gain $\eta =\bar{T_{D}}/\bar{T_{C}}$ can be calculated. Finally, from Equation (6), cooperation gain *G* achieved by the cooperative transmission can be obtained. By using Equations (4) and (5), we can get the network throughputs in DT and CT mode, i.e., *S*_D_ and *S*_C_.

## 6. Performance Evaluation

In this section, we simulate the performance of the proposed ORS-CMAC using C++ programming language, and compare the simulation results with the theoretical results to verify the effectiveness of the theoretical model. We mainly investigate the impact of the distance *d* between S and D, the total number of the links *N*_L_ in the network, and helper density λ_H_ on the network performance, i.e., the numbers of concurrent transmission links in DT and CT (i.e., *N*_D_ and *N*_C_), the spatial frequency reuse gain *ρ*, spatial diversity gain *η*, cooperation gain *G*, and network throughput.

### 6.1. Simulation Environment

In the simulation, we consider a circular region with the radius *R*_max_ = 2000 m. To obtain the upper bound of the network throughput, we let the transmission range equal the interference range, i.e., $R_{CT}^{I}=R_{DT}^{I}=R_{1}$. According to IEEE 802.11b, the network can support data rates of 1, 2, 5.5, and 11 Mbps. The corresponding transmission ranges are *R*_1_ = 100 m, *R*_2_ = 74.7 m, *R*_3_ = 67.1 m, and *R*_4_ = 48.2 m on the condition that the path loss exponent is 3 and bit-error-rate (BER) is lower than 10^−5^ [3]. Other simulation parameters are listed in Table 3. As shown in a previous study [37], the probability of selecting only one helper is 97.5055% when *k* = 4 and *M* = 3 if there are 25 potential helpers. Even if the helper density is 0.008 nodes/m^2^, the maximum number of the helpers is only 25. Therefore, we adopt *k* = 4 and *M* = 3 in the CHS process. The simulation results are obtained by averaging over 2000 randomly-generated network topologies. According to ORS-CMAC, if the distance *d* between S and D is lower than *R*_3_, direct transmissions are used for all links. Therefore, we only consider the case with *d* > *R*_3_. That is to say, we only focus on the performance differentiation between DT and CT. Therefore, the distance *d* in the simulation is set between 68 m and 100 m, which is larger than the transmission range of *r*_SD_ = 5.5 Mbps (i.e., *R*_3_). By default, *N*_L_ = 300, λ_H_ = 0.003 nodes/m^2^, and *d* = 70 m or 80 m.

### 6.2. Impact of d on Network Performance

**Figure 12:**
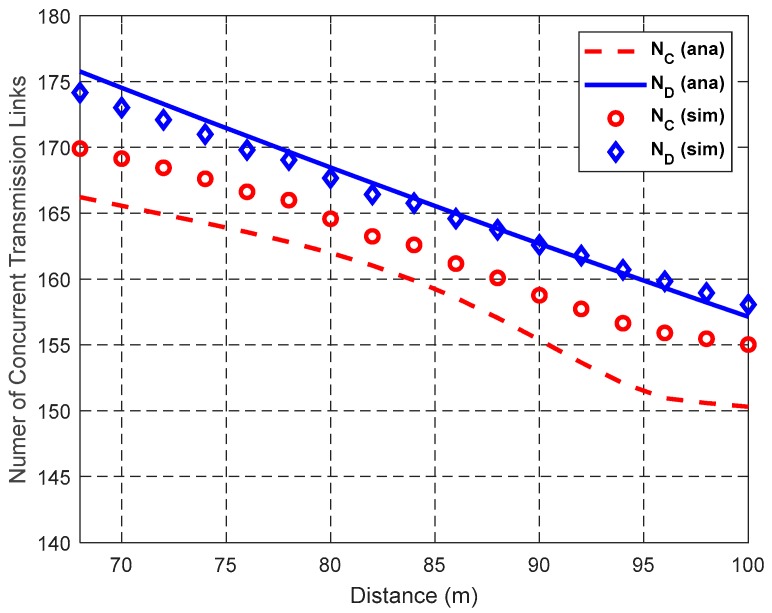
Impact of *d* on the number of concurrent transmission links.

**Figure 13:**
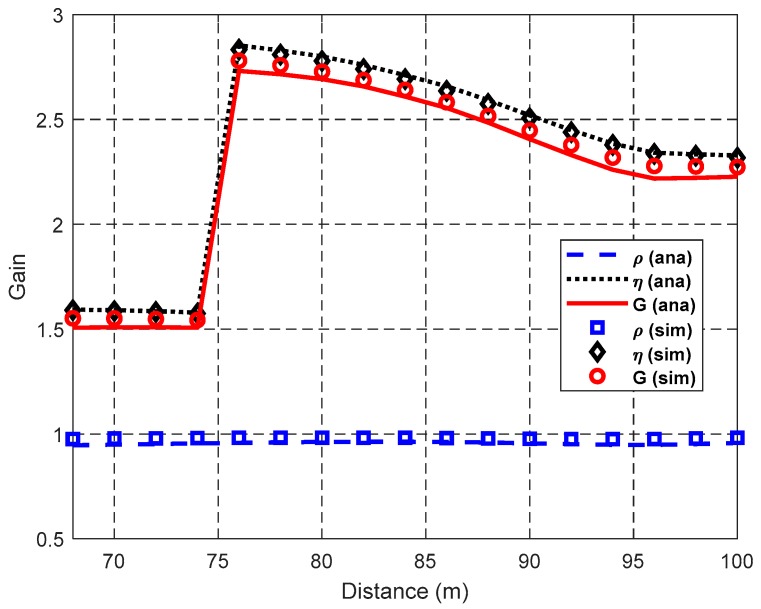
Impact of *d* on gain.

**Figure 14:**
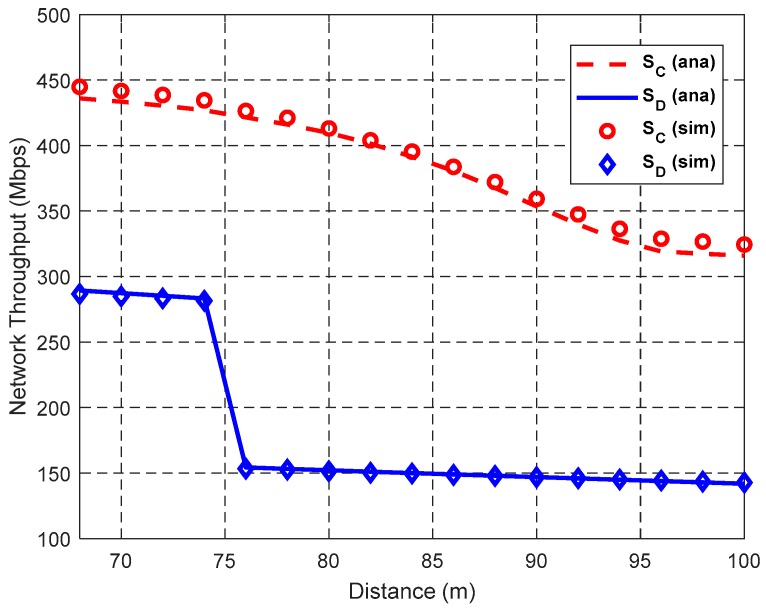
Impact of *d* on network throughput.

Figure 12, Figure 13 and Figure 14 show the impact of the distance *d* between S and D on the network performance, i.e., the numbers of concurrent transmission links in DT and CT (i.e., *N*_D_ and *N*_C_), the spatial frequency reuse gain *ρ*, spatial diversity gain *η*, cooperation gain *G*, and network throughput, when *N*_L_ = 300 and λ_H_ = 0.003 nodes/m^2^. It can be seen from the figures that the simulation results coincide with the theoretical results. The gaps between simulation results and theoretical results are due to the conservative estimate of the elliptical region model, which may block possible concurrent transmission links.

Figure 12 shows the impact of distance *d* between S and D on the number of concurrent transmission links in DT and CT of ORS-CMAC. Both the number of concurrent transmission links in DT, i.e., *N*_D_, and the number of concurrent transmission links in CT, i.e., *N*_C_, decrease with the increase of *d*. This is because an increase of *d* causes the enlarged IR as Equations (8), (9), (20), and (21) show, which increases the interference probability, and thus decreases the expected number of concurrent transmission links. It is obvious that the number of concurrent transmission links in CT is less than that in DT, i.e., *N*_C_ ≤ *N*_D_, due to the enlarged IR introduced by the helper. For example, when *d* = 70 m, the IRs of the direct transmission link and cooperative transmission link are 7.2099 × 10^4^ m^2^ and 8.2802 × 10^4^ m^2^, respectively. In most cases, the numbers of concurrent transmission links (i.e., *N*_C_ and *N*_D_) in the theoretical results are smaller than those in the simulation results with the same *d*. This is because we ignore the concurrent links that exist inside the LIR of other links and outside the NIR of other links for DT, as shown in Figure 8, and those inside the CLIR and outside the CNIR for CT, as shown in Figure 9. As shown in Figure 12, these differences increase with the augmentation of *d* because the area sizes inside LIR and CLIR and outside NIR and CNIR also increase with the augmentation of *d*.

It can be seen from Figure 13 that SFRG *ρ* is stable with the increase of *d* because the decreasing rates of *N*_C_ and *N*_D_ are almost the same, as Figure 12 shows. However, SDG *η* decreases with the increase of *d* when *R*_2_ < *d* < *R*_1_ and *R*_3_ < *d* < *R*_2_. This is due to the fact that the area of cooperation region A_ij_ decreases and then the probability of using the helper with a higher equivalent cooperative transmission rate decreases. These are also caused by cooperation gain *G* and SDG *η* having a similar trend. As described in Section 3, the direct transmission rates are *r*_SD_ = *r*_1_ = 1 Mbps when *R*_2_ < *d* < *R*_1_ and *r*_SD_ = *r*_2_ = 2 Mbps when *R*_3_ < *d* < *R*_2_, respectively. Thus, the SDG *η* and cooperation gain *G* when *R*_2_ < *d* < *R*_1_ are larger than those when *R*_3_ < *d* < *R*_2_. There is a rapid change of SDG *η*, which results in the same rapid change of cooperation gain *G* when *d* is around 75 m, because the boundary of using the transmission rates *r*_1_ and *r*_2_ for data packet transmissions is *R*_2_ = 74.7 m. When the data packet is transmitted with the lower transmission rate *r*_1_ instead of *r*_2_, $\bar{T_{D}}$ increases greatly because of this transmission rate reduction for DT and $\bar{T_{C}}$ increases much slower, thus increasing their quotient *η* suddenly. After that, with the growth of *d*, the SDG *η* decreases because $\bar{T_{D}}$ almost does not change with the same *r*_SD_, and meanwhile $\bar{T_{C}}$ increases a bit because the probability of employing the cooperative transmission *P_s-ij_* decreases with the area decrease of A_ij_.

As shown in Figure 14, the network throughput decreases with the increase of *d* because the IRs of the links increase and the number of concurrent transmission links decreases as *d* increases. Furthermore, the probability of using the highest cooperative transmission rate decreases, because the area of A_44_ decreases as *d* increases. When *d* ≤ *R*_3_ = 67.1 m, *S*_D_ and *S*_C_ are the same because all links adopt DT mode to transmit data packets. However, when *d* > *R*_3_, in spite of *N*_C_ ≤ *N*_D_, the network throughput in CT is larger than that in DT. This is because the SDG *η* achieved by cooperative transmissions can compensate for the reduction of SFRG *ρ*, thus improving the network throughput. When *d* is around 75 m, the rapid decrease of *S*_D_ is also caused by the *r*_SD_‘s transformation from *r*_2_ to *r*_1_.

### 6.3. Impact of N_L_ on Network Performance

**Figure 15:**
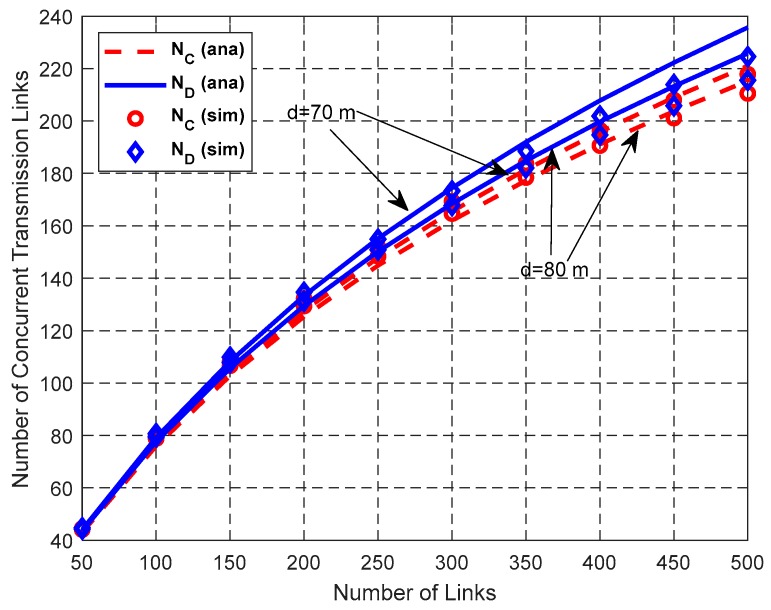
Impact of *N*_L_ on the number of concurrent transmission links.

**Figure 16:**
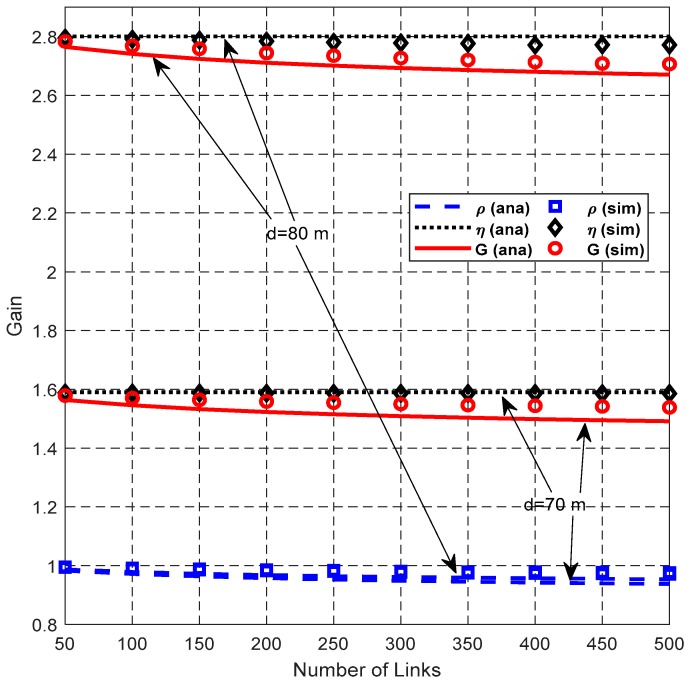
Impact of *N*_L_ on gain.

**Figure 17:**
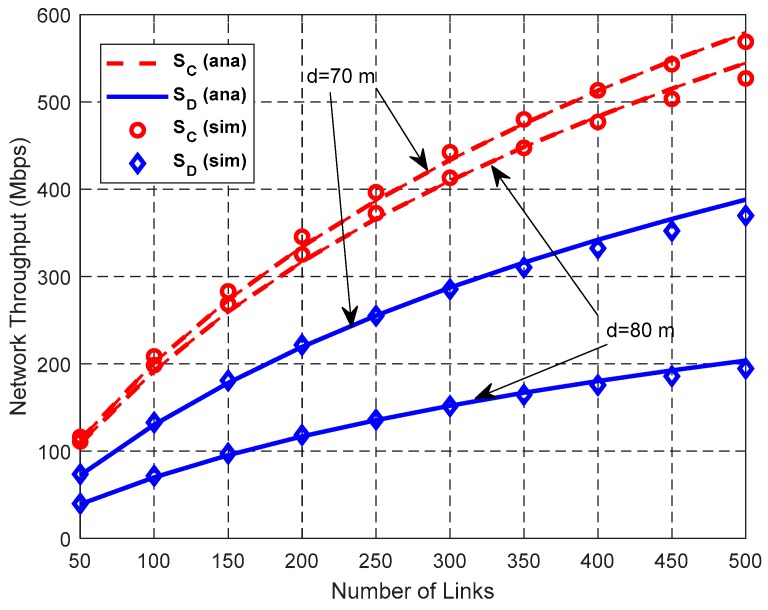
Impact of *N*_L_ on network throughput.

Figure 15, Figure 16 and Figure 17 show the impact of *N*_L_ on network performance when *λ*_H_ = 0.003 nodes/m^2^, and *d* = 70 m or 80 m.

As shown in Figure 15, both *N*_D_ and *N*_C_ increase with the increase of the total number of links *N*_L_ and *N*_D_ > *N*_C_. According to the random scheduling scheme, *P*(*k*, *n*) decreases with the increase of *n* for the same *k*. Therefore, the interference-free probability between the newly-added link and the already-existing links that can be scheduled concurrently decreases, which leads to the decrease of the increasing rate of the number of concurrent transmission links.

Figure 16 shows that the SDG *η* is stable with the increase of *N*_L_. However, SFRG *ρ* and cooperation gain *G* decrease slightly. This is because the adverse effect of the enlarged IR introduced by the helper is more serious as *N*_L_ increases. SFRG *ρ* with *d* = 80 m is only slightly smaller than that with *d* = 70 m, because the difference between *N*_D_ and *N*_C_ is relatively too small compared to the values of *N*_D_ and *N*_C_.

Figure 17 illustrates the relationship between network throughput and the total number of links *N*_L_. Both the network throughput *S*_D_ in DT mode and network throughput *S*_C_ in CT mode increase with the increase of *N*_L_. CT mode has higher network throughput than DT mode. This is because although SFRG *ρ* of CT decreases due to its enlarged IR, its SDG *η* increases faster than SFRG *ρ* and compensates for the reduction of SFRG *ρ*, thus improving the network throughput in the whole network.

### 6.4. Impact of λ_H_ on Network Performance

**Figure 18:**
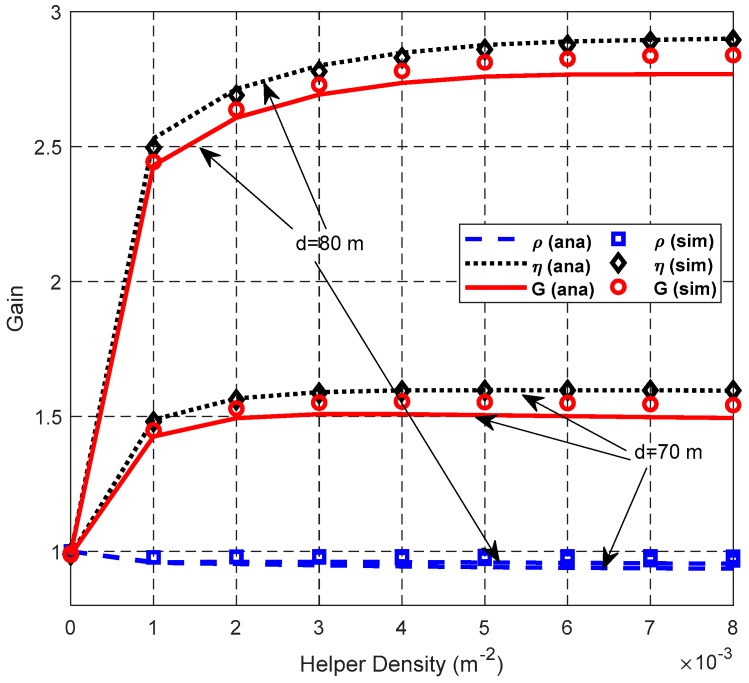
Impact of λ_H_ on gain.

**Figure 19:**
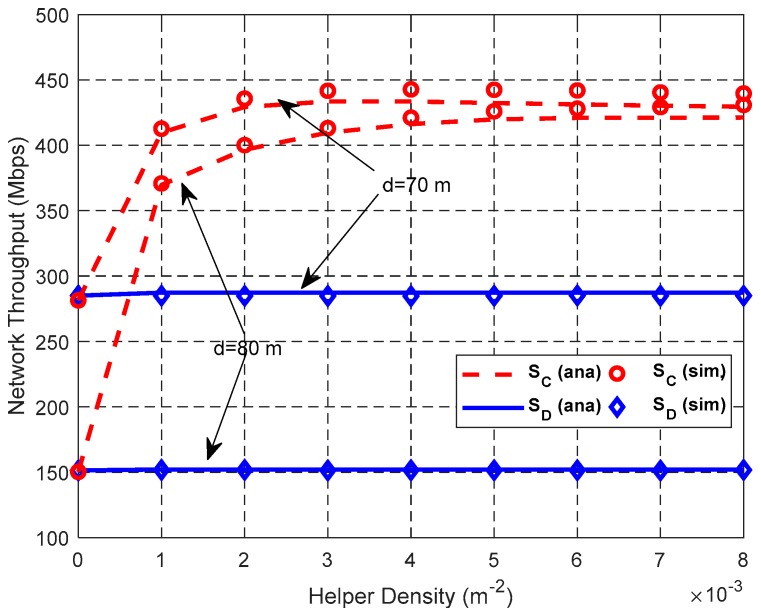
Impact of λ_H_ on network throughput.

Figure 18 and Figure 19 show the impact of helper density λ_H_ on the network performance with *N*_L_ = 300, and *d* = 70 or 80 m.

Figure 18 shows that the SFRG *ρ* decreases slightly with the increase of λ_H_ because the expected value of the largest Y-coordinate of the helper increases as λ_H_ increases, which increases the IR of the cooperative transmission link. The SDG *η* increases with the increase of λ_H_ and then decreases slightly when *d* = 70 m. This is because the probability that uses a higher equivalent cooperative transmission rate increases as λ_H_ increases. However, the probability of selecting only one helper decreases when the number of the helpers in a specific area becomes larger. Based on these, cooperation gain *G* retains a similar trend with the SDG *η*. The slope of the SDG *η* with the small helper density λ_H_ is larger than that with the large helper density, because at small helper densities, both the probability of using the cooperative transmission and the probability of using the cooperative transmission with higher equivalent cooperative transmission rate increases greatly with the growth of the number of helpers existing in the associated area A_ij_. When λ_H_ becomes large enough, the probability of employing the cooperative transmission with a higher equivalent cooperative transmission rate reaches close to the maximum value, and this results in the very small increments of SDG *η*.

Figure 19 shows that with the increase of λ_H_, the network throughput of DT *S*_D_ is stable, and the network throughput of CT *S*_C_ increases and then decreases slightly. As expected, the probability that employs a higher equivalent cooperative transmission rate increases as λ_H_ increases, thus increasing the network throughput in CT mode. However, if λ_H_ > 0.004 nodes/m^2^ and *d* = 70 m, the network throughput of CT decreases slightly as λ_H_ increases. This is because the number of helpers in the same A_ij_ increases with the increase of λ_H_, and thus decreases the success probability of selecting the optimal helper and increases the transmission time of a data packet.

## 7. Conclusions

A cooperative MAC protocol with optimal relay selection is proposed for large scale wireless networks in this paper. A priority differentiation scheme is adopted to rapidly select the helpers with the highest equivalent cooperative transmission rate for minimizing the IR, and *k*-CR scheme is employed to promptly select one optimal helper to participate in the cooperative transmission. To evaluate the protocol performance from the perspective of the whole network, we firstly construct the interference range models of DT link and CT link for each sender–recipient pair, and thus obtain the spatial frequency reuse gain. According to the packet transmission process in the proposed protocol, the average packet transmission times in DT mode and CT mode are derived, and thus the spatial diversity gain is obtained. Finally, the cooperation gain and network throughput are obtained. The effectiveness of the analytical model is validated by the simulation results. It is obvious that cooperative transmissions decrease the spatial frequency reuse gain *ρ* due to the enlarged IR, and the spatial diversity gain *η* remedies the reduction of *ρ*, thus improving the cooperation gain *G* and network throughput.

## Figures and Tables

**Table 1:** Relationship between data rate combination, priority level, cooperation rate, and corresponding minislot.

Data Rate Combination (*r*_SH_, *r*_HD_) (Mbps)	Priority Level	Cooperative Transmission rate	Corresponding Minislot
(11, 11)	*G*_R1_	*r*_C1_	1
(5.5, 11) or (11, 5.5)	*G*_R2_	*r*_C2_	2
(5.5, 5.5)	*G*_R3_	*r*_C3_	3
(2, 11) or (11, 2)	*G*_R4_	*r*_C4_	4
(2, 5.5) or (5.5, 2)	*G*_R5_	*r*_C5_	5

**Table 2:** Relationship between data rate combination and corresponding area.

Data Rate Combination (*r*_SH_, *r*_HD_) (Mbps)	Corresponding Area A_ij_
(11, 11)	A_44_
(5.5, 11) or (11, 5.5)	A_34_
(5.5, 5.5)	A_33_
(2, 11) or (11, 2)	A_24_
(2, 5.5) or (5.5, 2)	A_23_

**Table 3:** Simulation parameters.

Parameter	Value
MAC header	272 bits
PHY header	192 bits
RTS	160 bits
CTS/ACK/HTS	112 bits
*k*, *M*	4, 3
*L*_PKT_	1024 bytes
SIFS/τ	10 μs

## References

[1] Zhuang W., Zhou Y. (2013). A Survey of Cooperative MAC Protocols for Mobile Communication Networks. J. Internet Technol..

[2] Ju P., Song W., Zhou D. (2013). Survey on cooperative medium access control protocols. IET Commun..

[3] Liu P., Tao Z., Narayyanan S., Korakis T., Panwar S.S. (2007). CoopMAC: A Cooperative MAC for Wireless LANs. IEEE J. Sel. Areas Commun..

[4] Hunter T.E., Nosratinia A. (2006). Diversity through coded cooperation. IEEE Trans. Wirel. Commun..

[5] Kramer G., Gastpar M., Gupta P. (2005). Cooperative strategies and capacity theorems for relay networks. IEEE Trans. Inf. Theory.

[6] Laneman J.N., Tes D.N.C., Wornell G.W. (2004). Cooperative diversity in wireless networks: Efficient protocols and outage behavior. IEEE Trans. Inf. Theory.

[7] Saderk A.K., Su W., Liu K.J.R. (2007). Multinode cooperative communications in wireless networks. IEEE Trans. Signal Process..

[8] Zhou Y., Zhuang W. (2015). Throughput Analysis of Cooperative Communication in Wireless Ad Hoc Networks with Frequency Reuse. IEEE Trans. Wirel. Commun..

[9] Cai L.X., Cai L., Shen X., Mark J.W. (2010). REX: A random exclusive region based scheduling scheme for mmWave WPANs with directional antenna. IEEE Trans. Wirel. Commun..

[10] Gomez-Cuba F., Asorey-Cacheda R., Gonzalez-Castano F. (2012). A Survey on Cooperative Diversity for Wireless Networks. IEEE Commun. Surv. Tutor..

[11] Zhou T., Sharif H., Hempel M., Mahasukhon P., Wang W., Ma T. (2011). A Novel Adaptive Distributed Cooperative Relaying MAC Protocol for Vehicular Networks. IEEE J. Sel. Areas Commun..

[12] Zhu H., Cao G. (2006). rDCF: A Relay-Enabled Medium Access Control Protocol for Wireless Ad Hoc Networks. IEEE Trans. Mobile Comput..

[13] Bharati S., Zhuang W. (2013). CAH-MAC: Cooperative ADHOC MAC for Vehicular Networks. IEEE J. Sel. Areas Commun..

[14] Wang X., Li J. (2014). Network Coding Aware Cooperative MAC Protocol for Wireless Ad Hoc Networks. IEEE Trans. Parallel Distrib. Syst..

[15] Adam H., Yanmaz E., Bettstetter C. (2014). Medium Access with Adaptive Relay Selection in Cooperative Wireless Networks. IEEE Trans. Mobile Comput..

[16] Jibukumar M.G., Datta R., Biswas P.K. (2010). CoopMACA: A cooperative MAC protocol using packet aggregation. Wireless Netw..

[17] Khalid M., Wang Y., Ra I., Sankar R. (2011). Two-Relay-Based Cooperative MAC Protocol for Wireless Ad Hoc Networks. IEEE Trans. Veh. Technol..

[18] Zhou Y., Liu J., Zheng L., Zhai C., Chen H. (2011). Link-Utility-Based Cooperative MAC Protocol for Wireless Multi-hop Networks. IEEE Trans. Wirel. Commun..

[19] Shan H., Cheng H.T., Zhuang W. (2011). Cross-Layer Cooperative MAC Protocol in Distributed Wireless Networks. IEEE Trans. Wirel. Commun..

[20] Fasolo E., Rossetto F., Zorzi M. Network coding meets MIMO. Proceedings of the 2008 4th Workshop on Network Coding, Theory and Applications.

[21] Liu P., Nie C., Korakis T., Erkip E., Panwar S., Verde F., Scaglione A. (2012). STiCMAC: A MAC Protocol for Robust Space-Time Coding in Cooperative Wireless LANs. IEEE Trans. Wirel. Commun..

[22] Kim Y., Baccelli F., Veciana G. (2014). Spatial Reuse and Fairness of Ad Hoc Networks with Channel-Aware CSMA Protocols. IEEE Trans. Inf. Theory.

[23] Alawieh B., Zhang Y., Assi C., Mouftah H. (2009). Improving spatial reuse in multihop wireless networks–A survey. IEEE Commun. Surv. Tutor..

[24] Zhu J., Guo X., Yang L.L., Conner W.S. Leveraging spatial reuse in 802.11 mesh networks with enhanced physical carrier sensing. Proceedings of the IEEE International Conference Communications (ICC).

[25] Zhai H., Fang Y. Physical carrier sensing and spatial reuse in multirate and multihop wireless ad hoc networks. Proceedings of the 25th IEEE International Conference on Computer Communications (INFOCOM 2006).

[26] Zhang X., Zhang Y., Yan F., Vasilakos A.V. (2015). Interference-based topology control algorithm for delay-constrained mobile ad hoc networks. IEEE Trans. Mobile Comput..

[27] Wu Y., Hu Y., Su Y., Yu N., Feng R. (2018). Topology control for minimizing interference with delay constraints in an ad hoc network. J. Parallel Distrib. Comput..

[28] Zhang X.M., Yan L., Zhang H., Sung D.K. (2019). A concurrent transmission based broadcast scheme for urban VANETs. IEEE Trans. Mobile Comput..

[29] Soleimanpour-Moghadam M., Askarizadeh M., Talebi S., Esmaeili S. (2019). Low complexity green cooperative cognitive radio network with superior performance. IEEE Syst. J..

[30] Zhu Y., Zheng H. (2008). Understanding the Impact of Interference on Collaborative Relays. IEEE Trans. Mobile Comput..

[31] Bianchi G. (2000). Performance analysis of the IEEE 802.11 distributed coordination function. IEEE J. Sel. Areas Commun..

[32] Liu K., Chang X., Liu F., Wang X., Vasilakos A.V. (2015). A cooperative MAC protocol with rapid relay selection for wireless ad hoc networks. Comput. Netw..

[33] Li Y., Liu K., Liu F. (2013). CRP-CMAC: A priority-differentiated cooperative MAC protocol with contention resolution for multihop wireless networks. KSII Trans. Internet and Inf. Syst..

[34] Zhou Y., Zhuang W. Beneficial cooperation ratio in multi-hop wireless ad hoc networks. Proceedings of the IEEE INFOCOM.

[35] Cao B., Feng G., Li Y., Wang C. (2014). Cooperative Media Access Control with Optimal Relay Selection in Error-prone Wireless Network. IEEE Trans. Veh. Technol..

[36] Chang X., Liu K., Liu F. (2015). Performance analysis of a *k*-round contention resolution scheme for WLANs. Electron. Lett..

[37] Chang X., Liu K., Xu Z., Liu F. A relay-optimized cooperative MAC protocol for mobile ad hoc networks. Proceedings of the IEEE 17th International Conference on Computational Science and Engineering (CSE 2014).

